# Notes and Notices

**Published:** 1892-08

**Authors:** 


### NOTES AND NOTICES.

Horlick's Malted Milk is daily coming into extended use and prominence as a food for infants and for nursing mothers. Infants thrive better on it than on cow's milk, and it is often retained and assimilated where everything else is rejected.

It is being used not only by physicians in their practice but in most of the principal asylums and hospitals for children all over the United States, and seems to be giving excellent satisfaction everywhere. The factory near Racine, Wis., is located in the finest farming district of the Northwest, and is surrounded by everything favorable to the production of a perfect infant food.

Dr. Skinner begs to inform his patients and their friends that he takes his annual holiday this year from the first of August to the end of September, and that he can see no patients after Friday, 29th July. Letters directed to Dr. S., Mybster Inn, by Watten, Caithness, N. B., will be replied to in course of post. Telegrams, though objectionable, may be directed, "Skinner, Mybster, Georgemas, Caithness," four miles, which will be replied to by letter.

25 Somerset Street, London, W., June, 1892.

